# Roles of Macrophage Migration Inhibitory Factor (MIF) Signaling Pathway in Oncovirus Infection and Virus-Associated Cancers

**DOI:** 10.3390/v17121582

**Published:** 2025-12-05

**Authors:** Jiaojiao Fan, Victor Ryu, Zhiqiang Qin, Lu Dai

**Affiliations:** Department of Pathology, Winthrop P. Rockefeller Cancer Institute, University of Arkansas for Medical Sciences, 4301 W Markham St, Little Rock, AR 72205, USA; jfan@uams.edu (J.F.); vryu@uams.edu (V.R.); zqin@uams.edu (Z.Q.)

**Keywords:** MIF, oncovirus, HBV, HPV, EBV, HCMV, KSHV

## Abstract

Approximately 12% of all human cancers are caused by oncoviruses. Macrophage migration inhibitory factor (MIF) signaling activation has been found closely related to many cancer cell malignant behaviors and infectious disease progression. However, its role in virus-associated cancers or how oncoviruses may regulate MIF signaling activities remains largely unknown. In the current review, we summarize recent findings about the oncovirus activation of MIF signaling pathways, their functional roles in viral oncogenesis, and the development of MIF-targeted therapies. We also discuss future directions in this interesting field.

## 1. Introduction

The macrophage migration inhibitory factor (MIF) is a highly conserved, multifunctional cytokine that regulates the movement of immune cells. While its original function was primarily recognized as a blocker of immune cell migration, MIF is now understood to have a broader role in immunoregulation and tissue homeostasis [1,2]. MIF is produced by various cell types in the body, including immune cells like macrophages and T cells, as well as non-immune cells. It is rapidly released in response to microbial products, inflammatory signals, and cellular stress [1,2]. Structurally, MIF is a homotrimeric protein that, when active, functions as an enzyme interacting with receptors and activating downstream signaling pathways. For example, it acts as a tautomerase, converting tautomers such as keto-phenylpyruvate to enol-phenylpyruvate, a process important for metabolism and cellular stress responses. Additionally, MIF exhibits oxidoreductase activity, facilitated by its CXXC motif, enabling it to neutralize harmful oxidants and reduce oxidative stress [3].

**Figure 1:**
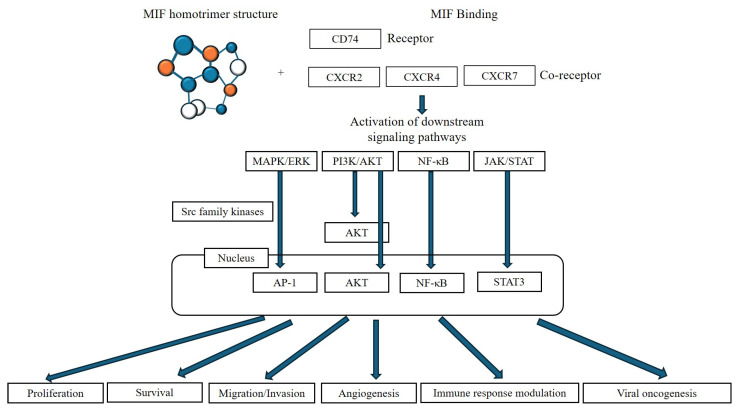
MIF signaling pathways and regulation of cellular functions in cancer cells.

The MIF signaling pathway begins with MIF binding to its primary receptor, CD74, on the plasma membrane. In many cases, it also involves chemokine receptors like CXCR2, CXCR4, and CXCR7 [3]. These receptors and co-receptors activate several key intracellular pathways, including MAPK/ERK, PI3K/AKT, NF-κB, and JAK/STAT pathways, which regulate essential cellular processes such as proliferation, migration, survival, angiogenesis, and apoptosis resistance [3,4,5,6] (Figure 1). MIF can also be internalized by cells via endocytosis, where it interacts with cellular factors like Jun Activation Domain-binding Protein 1 (JAB1) and COP9 signalosome subunit 5 (CSN5), influencing various cellular activities, including gene regulation, metabolism, immune responses, and tumor progression [3].

In cancer biology, MIF and its downstream signaling pathways promote cancer cell survival, growth, and immune evasion. MIF supports sustained proliferation, survival, angiogenesis, invasion, and metastasis [7,8]. Elevated MIF expression is observed in many tumor types, contributing to cancer progression and poor prognosis. Mechanistically, MIF can enhance epithelial-to-mesenchymal transition (EMT), regulate tumor suppressor genes, and create hypoxic and inflammatory tumor microenvironments that promote tumor progression and immune evasion [6,7,8]. Consequently, MIF and its receptors/co-receptors, particularly CD74, are considered attractive therapeutic targets for the development of novel anticancer therapies [8,9].

Approximately 12% of human cancers are associated with oncoviral infections, including Epstein–Barr virus (EBV), hepatitis B and C viruses (HBV and HCV), human cytomegalovirus (HCMV), human papillomavirus (HPV), and Kaposi’s sarcoma-associated herpesvirus (KSHV) [10]. While much is known about MIF signaling in conventional cancers, there is limited data on the role of MIF signaling activation in virus-associated cancers, including how oncoviruses may regulate MIF signaling. This review summarizes recent findings on the regulation of MIF signaling by oncoviruses, its role in viral oncogenesis, and the potential for MIF-targeted therapies.

## 2. MIF and Epstein–Barr Virus (EBV)

EBV, an oncogenic herpesvirus, is associated with various malignancies, such as Hodgkin’s lymphoma, Burkitt’s lymphoma, stomach cancer, and nasopharyngeal carcinoma (NPC). EBV encodes several latent and lytic proteins that potentiate NF-κB activation and stimulate the secretion of inflammatory factors in NPC [11,12]. Overexpression of MIF has been observed in EBV-positive NPC tumor tissues and immune lymphocytes from Hodgkin’s lymphoma patients [13,14]. As a downstream molecule of MIF, IL-8 is also elevated in NPC tumor tissues and poorly differentiated NPC cell lines [15]. Mechanistic studies show that MIF knockdown, via RNA interference (RNAi) or NF-κB inhibitor Parthenolide, reduces IL-8 expression and inhibits the growth of tumor spheres. MIF overexpression promotes the survival of EBV-transformed B cells by suppressing apoptosis signal-regulating kinase 1 (ASK1)/JNK-mediated transcriptional activation of p63 (TAp63), which leads to mitochondria-dependent apoptosis [16]. Additionally, MIF modulates tumor-associated macrophages (TAMs) and recruits regulatory myeloid populations, contributing to immune evasion in EBV-associated tumors [17]. A significant positive correlation between EBV-encoded EBER1 levels and MIF expression has been found in NPC tissues. These findings suggest that MIF plays a crucial role in the pathogenesis of EBV-associated malignancies.

## 3. MIF and Hepatitis B and C Virus (HBV/HCV)

HBV and HCV infections are major risk factors for viral hepatitis, liver fibrosis, and hepatocellular carcinoma (HCC). Elevated serum MIF levels correlate with liver fibrosis and poor prognosis in HBV-related HCC patients [18,19]. The HBV-encoded regulatory protein HBx binds to MIF, playing a crucial role in counteracting apoptosis in HCC cells [20]. MIF is a key cytokine that regulates the immunosuppressive tumor microenvironment and disrupts normal T cell function. In an HBV-positive HCC mouse model, inhibition of MIF expression by sulforaphane (a compound derived from traditional Chinese medicine) reversed immune tolerance by promoting macrophage polarization towards the M1 phenotype and restoring the balance of immune regulatory cells, such as Tregs and Th17 cells [21].

Furthermore, higher frequencies of MIF polymorphisms, particularly the MIF-173 G/C variant, have been observed in Iranian patients with chronic HBV, suggesting that certain MIF polymorphisms may influence susceptibility to chronic hepatitis B infection [22]. Consistent with these findings, a study of Egyptian patients with HCC revealed a significantly higher frequency of the MIF-173 G/C (rs755622) single-nucleotide polymorphism (SNP) compared to the control group [23]. Additional data also show that specific MIF polymorphisms are associated with disease severity and complications in HCV-induced fibrosis, with effects that are stage- and context-dependent [24]. Taken together, these findings support the role of MIF in the development of HBV/HCV-induced liver cancer.

## 4. MIF and Human Cytomegalovirus (HCMV)

HCMV infection is typically asymptomatic in healthy individuals but significantly increases mortality risk in immunocompromised patients and transplant recipients due to complications such as CMV hepatitis, cytomegalovirus retinitis, and CMV pneumonitis. Accumulation of MIF protein has been observed following HCMV infection of human foreskin fibroblasts, which is associated with activation of the NF-κB signaling pathway and subsequent secretion of pro-inflammatory cytokines [25]. The HCMV-encoded proteins IE1 and IE2 are responsible for upregulating pro-inflammatory cytokines and growth factors during viral replication [26]. However, the potential interactions between IE1/IE2 and MIF signaling remain to be elucidated.

## 5. MIF and Human Papillomavirus (HPV)

High-risk HPV types drive cervical and certain head and neck cancers primarily through the actions of the viral oncoproteins E6 and E7, which regulate NF-κB, p53, and pRb pathway activities [27]. Several studies have reported elevated MIF expression in HPV-positive cervical intraepithelial neoplasia and carcinoma [28]. The E6 and E7 oncoproteins appear to enhance MIF secretion by increasing lactate production (via the Warburg effect) and inducing hypoxia-inducible factor 1α (HIF-1α) expression. This contributes to immune evasion by inhibiting the initiation of immune responses and promoting immunosuppressive myeloid cell phenotypes [28]. Therefore, MIF acts as a major mediator amplifying HPV-driven oncogenic signaling and tumor microenvironment remodeling.

## 6. MIF and Human T-Lymphotropic Virus 1 (HTLV-1)

HTLV-1, a retrovirus of the human T-lymphotropic virus family, causes adult T cell leukemia/lymphoma (ATL) and HTLV-1-associated myelopathy/tropical spastic paraparesis (HAM/TSP). Data from transgenic mouse models show that abnormal MIF mRNA expression correlates with HTLV-1-induced tumorigenesis in vivo [29]. Elevated MIF expression has also been observed in various HTLV-1-infected human T cell lymphoma cell lines [30]. Functionally, overactivation of the MIF/CD74 axis promotes HTLV-1-associated tumorigenesis through NF-κB and ERK1/2-mediated cell proliferation [31].

## 7. MIF and Kaposi’s Sarcoma-Associated Herpesvirus (KSHV)

KSHV infection can cause several malignancies, including Multicentric Castleman’s disease (MCD), Kaposi’s sarcoma (KS), and primary effusion lymphoma (PEL). A study of KSHV infection among sex workers and women from the general population in Spain reported that KSHV DNA was detected in 2% of cervical samples from sex workers and in 1% of samples from women in the general population [32]. Another study documented oral co-infection of HPV and KSHV among HIV-infected individuals in Italy, particularly among men who have sex with men [33]. Our group was the first to report that the HPV-positive cervical cancer cell line SiHa is susceptible to KSHV latent infection [34]. Cytokine array analysis revealed that KSHV co-infection induces the production of several inflammatory cytokines and chemokines, including MIF, in SiHa cells. Furthermore, our data show that KSHV co-infection upregulates MIF and its receptors (e.g., CD74, CXCR2, CXCR4) both in vitro and in vivo [35].

However, currently no mechanistic studies are about how KSHV activates MIF signaling, especially which specific KSHV proteins or components are able to regulate MIF signaling in cancer cells. It would also be of interest to determine whether MIF and its receptors/co-receptors are similarly elevated in KS and PEL, and to elucidate their functional contributions to viral oncogenesis. Notably, several downstream signaling pathways regulated by MIF— including MAPK/ERK, PI3K/AKT, NF-κB, and HIF-1α/VEGF— have been reported to be highly activated in KSHV-infected tumor cells [36,37,38].

## 8. The Development of MIF-Targeted Therapy

Targeting MIF signaling for therapeutic purposes has emerged as a promising approach in oncology and virology, given its crucial role in tumor progression, viral persistence, and immunoregulation (Table 1), although current data are mostly based on oncovirus-negative cancers. Several early inhibitors targeting MIF include ISO-1, OXIM-11, and 4-iodo-6-phenylpyrimidine (4-IPP). These compounds deactivate the tautomerase activity of MIF by binding to its active site through distinct mechanisms. ISO-1 competitively inhibits MIF’s tautomerase activity by binding to its hydroxyphenyl group, thereby preventing interaction with the CD74 receptor complex and reducing activation of the NF-κB pathway [39]. ISO-1 has been reported to significantly decrease proliferation, migration, and invasion of human pancreatic cancer (PANC-1) cells in vitro. OXIM-11 interacts with MIF’s active site through hydrogen bonding and hydrophobic interactions via its carbonyl oxime scaffold [40]. In contrast, 4-IPP binds covalently to the MIF active site, irreversibly modifying the catalytic N-terminal Pro1 residue. This modification blocks MIF-dependent downstream signaling and suppresses cancer stem cell traits [41].

Another therapeutic strategy involves disrupting the interaction between MIF and its receptor CD74, along with associated chemokine co-receptors such as CXCR2, CXCR4, and CXCR7. Imalumab (BAX69) is a recombinant monoclonal antibody that specifically binds to the oxidized form of MIF (oxMIF) found in tumor tissues and inflammatory sites. By targeting oxMIF, Imalumab effectively neutralizes MIF activity [42]. In a phase I clinical study, Imalumab treatment demonstrated tolerable toxicity and early signs of disease stabilization in patients with advanced solid tumors. Interestingly, inhibition of the MIF–CD74 axis and its co-receptors may also modulate immune responses within the tumor microenvironment, influencing inflammation, angiogenesis, and potentially enhancing treatment responsiveness [43].

Additionally, compounds such as Ibudilast can bind non-competitively to a site adjacent to MIF’s active center, inducing conformational changes that inhibit both its enzymatic and inflammatory functions [44]. MIF overexpression has been documented in glioblastoma (GBM). One study reported that Ibudilast exhibited modest antiproliferative effects on patient-derived GBM cell lines; however, when combined with temozolomide (TMZ), it showed significant synergistic activity, resulting in cell cycle arrest and apoptosis. In a patient-derived xenograft (PDX) model, combined Ibudilast and TMZ treatment significantly prolonged overall survival [45]. Recently, dual-active inhibitors such as p425 and CPSI-1306 have been developed. These compounds disrupt MIF’s allosteric structure and block the formation of co-receptor complexes, demonstrating broad anticancer potential while maintaining favorable toxicity profiles [46].

## 9. Conclusions

In contrast to other conventional cancers, the roles of MIF signaling activation and its mediated functions in viral oncogenesis remain largely unexplored, hindering the development of MIF-targeted strategies for the prevention and treatment of these malignancies. Future studies should focus on several key areas: (1) molecular mechanisms underlying the activation of MIF signaling pathways by oncoviruses, including regulation of MIF expression (or some post-translational modifications) and secretion, its receptors/co-receptors, and downstream effectors; (2) impacts of MIF signaling activation on oncoviral gene expression, replication, and pathogenesis, including herpesviral lytic reactivation; (3) regulation of the tumor microenvironment by MIF signaling, for example, whether these oncoviruses manipulate MIF signaling to promote tumor immune evasion through similar or different mechanisms; (4) comparative analysis of MIF signaling activities between oncovirus-positive and -negative cancer cells, such as HBV-positive versus HBV-negative HCC or HPV-positive versus HPV-negative cervical cancer; (5) evaluation of MIF inhibitors, assessing the efficacy of different types of MIF-targeted compounds in virus-associated cancers to identify the most effective therapeutic agents, including their effects on viral gene expression and functions in cancer cells; (6) potential development of resistance to MIF-targeted therapies by oncoviruses through viral or host cellular mechanisms. In addition, it should be noted that elevated MIF levels or association with diseases including virus-associated cancer do not necessarily imply direct functional interactions or causality, which definitely require more experimental evidence.

Overall, accumulating evidence supports that MIF signaling pathways play a central role in the pathogenesis of many virus-associated malignancies, representing attractive targets for the development of novel therapeutic strategies.

## Figures and Tables

**Table 1:** Representative MIF inhibitors in development.

Inhibitor	Type	Target Site or Action	Functional Effects	Diseases	References
ISO-1	Small-molecule	Tautomerase MIF/CD74	Suppress tumor proliferation, migration and invasion	Pancreatic cancer	[39]
OXIM-1	Small-molecule	Bind to MIF active site by hydrogen bonds and hydrophobic interaction	Decrease NF-κB; reduce inflammation	Acute peritonitis	[40]
4-1PP (4-iodo-6-phenylpyrimidine)	Small-molecule	Bind covalently to MIF active site	MIF degradation; block NF-κB/P-TEFb complex formation	Osteosarcoma	[41]
Imalumab (BAX69)	Monoclonal antibody	oxMIF MIF/CD74	Neutralize MIF activity; suppress tumor growth and metastasis	Malignant solid tumor, mCRC, NSCLC, ovarian cancer	[42,43]
Ibudilast	Small-molecule	Non-competitively bind to adjacent site of MIF and cause conformation change	Downregulate MIF expression and functions	Neuronopathic pain, opioid withdrawal, acute opioid analgesia	[44,45]
P425/CPSI-1306	Dual-active inhibitor	Disrupt allosteric structure of MIF MIF/CD74	Inhibit tautomerase and biological properties of MIF	Inflammatory diseases	[46]

## Data Availability

No new data were created or analyzed in this study.
